# Donor Smoking Negatively Affects Donor and Recipient Renal Function following Living Donor Nephrectomy

**DOI:** 10.1155/2011/929263

**Published:** 2011-09-06

**Authors:** Jonathan Heldt, Robert Torrey, Daniel Han, Pedro Baron, Christopher Tenggardjaja, Justin McLarty, Tekisha Lindler, D. Duane Baldwin

**Affiliations:** ^1^Department of Urology, Loma Linda University Medical Center, Loma Linda, CA 92354, USA; ^2^Transplant Surgery, Loma Linda University Medical Center, Loma Linda, CA 92354, USA

## Abstract

*Background.* While tobacco use by a renal transplant recipient has been shown to negatively affect graft and patient survival, the effect of smoking on the part of the kidney donor remains unknown. *Methods.* 29 smoking donors (SD) and their recipients (SD-R) as well as 71 non-smoking donors (ND) and their recipients (ND-R) were retrospectively reviewed. Preoperative demographics and perioperative variables including serum creatinine (Cr) and glomerular filtration rate (GFR) were calculated and stratified by amount of tobacco exposure in pack-years. Clinical outcomes were analyzed with a Student's *t*-test, chi-square, and multiple linear regression analysis (*α* = 0.05). *Results.* At most recent followup, SD-R's had a significantly smaller percent decrease in postoperative Cr than ND-R's (−57% versus −81%; *P* = 0.015) and lower calculated GFR's (37.0 versus 53.0 mL/min per 1.73 m^2^; *P* < 0.001). SD's had a larger percent increase in Cr than ND's at most recent followup (57% versus 40%; *P* < 0.001), with active smokers having a larger increase than those who quit, although this difference was not statistically significant (68% versus 52%; *P* = 0.055). *Conclusions.* Use of tobacco by kidney donors is associated with decreased posttransplant renal function, although smoking cessation can improve outcomes. Kidneys from donors who smoke should be used with caution.

## 1. Introduction

Cigarette smoking is the leading preventable cause of death in the United States and is thought to be responsible for about one in five deaths annually, or approximately 438,000 deaths per year [1]. The effects of tobacco use on the body are widespread, affecting primarily the cardiovascular [2] and pulmonary [3, 4] systems while exerting carcinogenic effects in multiple organ systems [5–8]. As with other organs, the kidney is susceptible to the pathogenic effects of tobacco use [9]. During the last decade, a body of research has accumulated demonstrating the deleterious effects of recipient tobacco consumption upon graft function and patient outcomes [10–15]. However, the effect of cigarette smoking by the kidney donor has not previously been reported. The purpose of this study is to retrospectively compare renal function in donors with a history of tobacco use against donors who have never smoked as well as to evaluate their respective recipients' renal function and graft outcomes.

## 2. Materials and Methods

A retrospective chart and database review was performed on 100 hand-assisted laparoscopic donors and their 100 recipients at a single university hospital from February 2003 through June 2005, with 29 smoking donors (SD) and their recipients (SD-R) as well as 71 non-smoking donors (ND) and their recipients (ND-R). Demographic data including age, body mass index (BMI), sex, and ethnicity were obtained and evaluated for all patients. Donor creatinine (Cr) levels were evaluated at 1 week, 3 months, and 6 months while recipient Cr levels were evaluated at 1 month, 6 months, and at 6 month intervals thereafter. In addition, levels of the immunosuppressive drugs cyclosporine A and tacrolimus were also compared in recipients at 1 week, 3 months, and 6 months.

As dictated by our hospital's renal transplantation protocol, none of the recipients were active users of tobacco at the time of transplantation. Surgical technique for the SD and ND groups was identical, consisting of a hand-assisted laparoscopic donor nephrectomy in a 45-degree lateral decubitus position. Recipient surgical technique and immunosuppression regimen were identical for the SD-R and ND-R groups during the period of review. 

Patients were grouped based on the donor's history of tobacco use and then further stratified by total quantity and timing of tobacco exposure in pack-years. Absolute Cr levels (mg/dL) and percent changes in Cr were calculated for the donors and recipients at each of the different time intervals to assess renal function. In addition, glomerular filtration rates (GFR) in mL/min per 1.73 m^2^ were calculated for adult patients using the Modification of Diet in Renal Disease Study equation [16]. Recipients under the age of 18 were excluded from GFR calculations. Statistical analysis was performed using an unpaired two-tailed Student's *t*-test for continuous variables and a chi-square test with Yates correction for categorical variables. A multiple linear regression analysis was performed to control for confounding variables. Variables incorporated into the multivariate analysis included donor sex, age, race, BMI, and comorbidity status (diabetes and hypertension) as well as recipient sex, age, race, BMI, comorbidity status, tobacco use, and immunosuppressive drug levels. Kaplan-Meier survival curves for renal allograft survival with Mantel-Cox logrank analysis were constructed to evaluate median survival time of the grafts. Significance for all tests was established at *α* = 0.05.

## 3. Results

There were no statistically significant differences between the groups with respect to age, BMI, sex, or presence of significant comorbidities such as hypertension and diabetes (Table 1). No patients in either donor group had hypertension requiring medication or hospitalization, as this comorbid condition would preclude kidney donation at our institution. Ethnicity in the donor group was also evaluated, but due to a prevalence of Hispanic (43%) and Caucasian (46%) patients, the relative effects of African-American (7%) and Asian (2%) race could not be accurately assessed. Thirteen patients in the SD group (45%) were actively smoking at the time of surgery while the remaining 16 had a past history of previous tobacco use but had quit smoking by the time of donation. 

**Figure 1:**
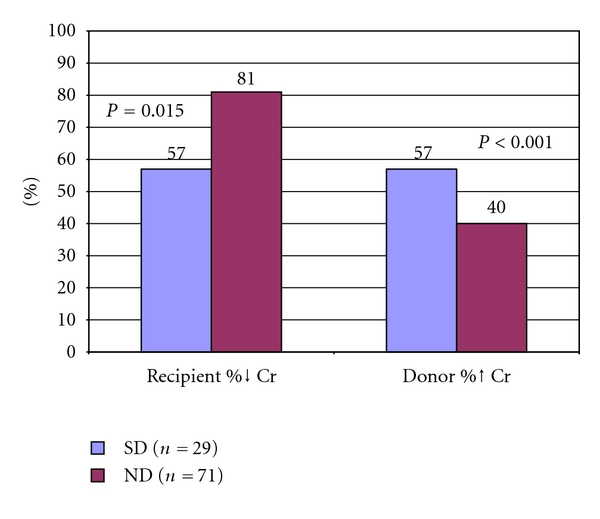
Comparison of percent change in creatinine (Cr) at most recent followup between recipients of kidneys from donors with a history of tobacco use (SD) and those with no history of tobacco use (ND) as well as the percent change in Cr of the donors.

**Figure 2:**
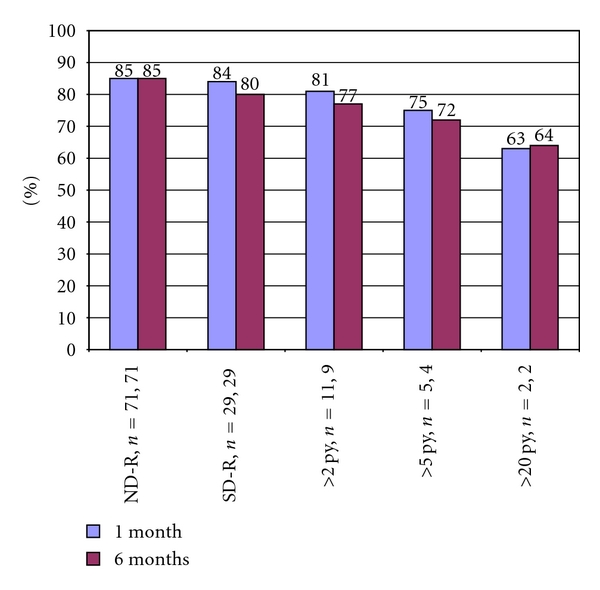
Comparison of percent decrease in creatinine (Cr) at 1 and 6 months between the recipients of renal allografts from living donors who have a history of tobacco use (SD-R) and donors with no history of tobacco use (ND-R) stratified by donor smoking exposure in pack-years (py).

**Figure 3:**
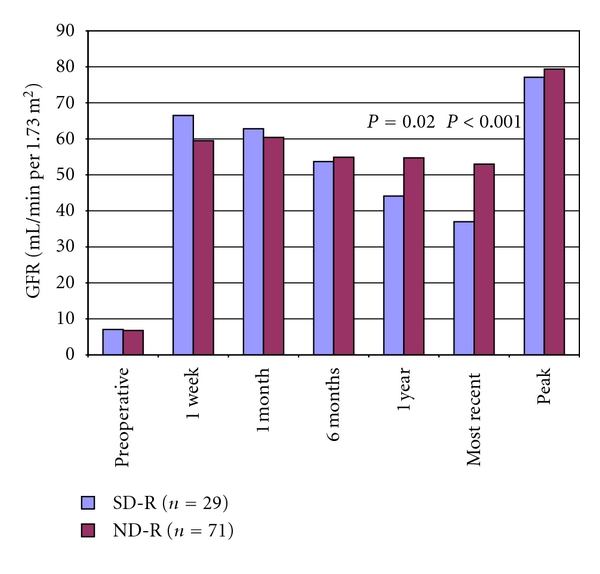
Comparison of calculated glomerular filtration rates in mL/min per 1.73 m^2^ between recipients of renal allografts from living donors who have a history of tobacco use (SD-R) and donors with no history of tobacco use (ND-R).

In regards to the recipients, multivariate analysis using a multiple linear regression model revealed that only the smoking status of the donor had a significant effect upon recipient percent change in Cr (*P* = 0.026). SD-R's had a smaller percent decline in their postoperative Cr when compared to ND-R's, although this was not significant at 1 or 6 months of followup. However, at most recent followup (mean of 40 months for SD-R's and 38 months for ND-R's; *P* = 0.38), there was a significantly smaller percent decline in Cr when comparing SD-R's to ND-R's (−57% versus −81%; *P* = 0.015; Figure 1). This effect of donor tobacco exposure was exhibited in a dose response manner, with progressively increasing levels of prior donor tobacco exposure resulting in progressively smaller improvements in recipient Cr (Figure 2). Comparing calculated GFR values between the recipient groups revealed that SD-R's had significantly lower GFR's at 1 year (44.1 versus 54.7 mL/min per 1.73 m^2^, respectively; *P* = 0.018) and at most recent followup (37.0 versus 53.0 mL/min per 1.73 m^2^, resp.; *P* < 0.001; Table 2, Figure 3).

In regards to graft survival in the recipients, the SD-R group had a higher rate of graft failure resulting in dialysis compared to the ND-R group at most recent followup (6/13 (46%) versus 5/30 patients (17%)), although this difference was not statistically significant. Of the 6 SD-R's with graft failure at this follow-up interval, 4 were due to acute cellular rejection while the remaining 2 instances were caused by chronic allograft nephropathy. In the ND-R group, 2 instances of graft failure were due to acute humoral rejection, 2 were due to acute cellular rejection, and the remaining instance was caused by chronic allograft nephropathy. Incidences of graft failure did have a significant impact on the observed percent change in Cr, with SD-R's undergoing rejection experiencing a significantly lower reduction in Cr than SD-R's with functioning allografts (8.5% versus 75.5%, resp.; *P* = 0.002). A similar trend was observed when comparing the effect of graft failure on changes in Cr within the ND-R group (69.6% for those experiencing allograft failure versus 82.0% for those with functioning allografts; *P* = 0.004). The observed differences in graft failure were not caused by variations in immunosuppression regimen, as levels of cyclosporine A and tacrolimus did not differ significantly between the two groups at any time interval compared. In addition, there were no differences between the SD and ND groups in other potentially confounding factors such as warm ischemia time (128.3 versus 132.6 seconds, resp.; *P* = 0.67), rate of zero antigen mismatch (10.3% versus 7.0%, resp.; *P* = 0.58), or share of delayed graft function (0% in both groups). 

**Figure 4:**
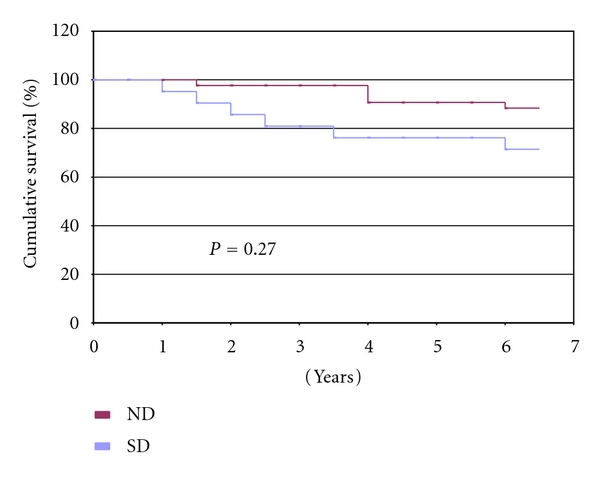
Kaplan Meier renal allograft survival curves for kidneys from living donors with a history of tobacco use (SD) and those with no history of tobacco use (ND). Statistical significance between mean survival times is assessed using the Mantel-Cox logrank test.

Kaplan Meier renal allograft survival curves for both groups are shown in Figure 4. While incidence of graft failure did differ between the groups, Mantel-Cox logrank analysis revealed no statistically significant difference in median survival time of the graft between the SD-R and ND-R groups (6.2 versus 6.6 years, resp.; *P* = 0.27; Figure 4).

**Figure 5:**
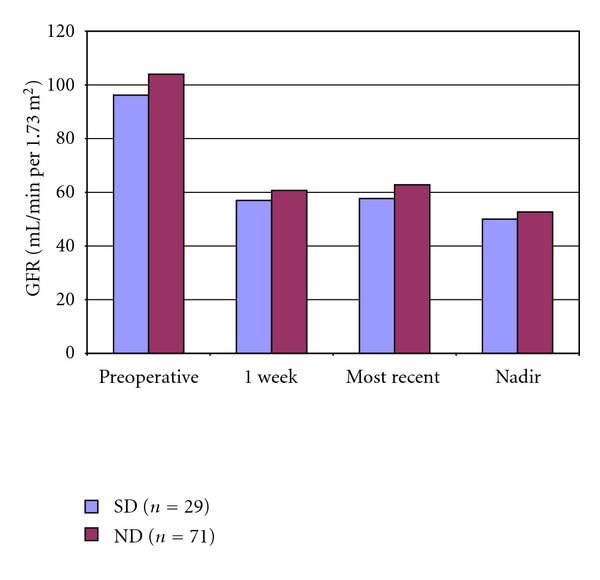
Comparison of calculated glomerular filtration rates in mL/min per 1.73 m^2^ between living kidney donors with a history of tobacco use (SD) and those with no history of tobacco use (ND).

Postoperative changes in donor Cr were also compared. At most recent followup (mean of 144 days for SDs and 143 days for NDs; *P* = 0.98), SDs demonstrated a greater percent change in Cr than NDs (57% versus 40%; *P* < 0.001; Figure 1). GFR was higher among NDs than SDs at all time intervals compared, although none of these differences reached statistical significance (Table 2, Figure 5). Within the SD group, current smokers had a larger percent increase in Cr at most recent followup than those who had quit smoking by the time of donation, although this difference fell just short of significance (68% versus 52%; *P* = 0.055). No donors in either group developed postoperative hypertension requiring medication or hospitalization at most recent followup.

## 4. Discussion

Tobacco use has been demonstrated to have pathologic effects on the kidney. Patients who smoke experience increased sympathetic nervous activity, leading to hypertension, hyperfiltration, albuminuria, and proximal tubular function damage, while also having a higher prevalence of chronic renal disease [9, 17]. Patients exposed to tobacco also have considerable changes in endothelial cell ultrastructure which increase the risk of atherosclerosis, hyperactive platelets which increase the chance of thrombogenesis, and an altered immune system leading to immune-mediated renal diseases [9, 17]. 

The deleterious effects of tobacco use on the part of the renal allograft recipient have been clearly defined. In one study, recipients had a 2.1-times greater risk of poor long-term graft outcomes if they had a history of smoking prior to transplantation (*P* < 0.001) [11]. Additionally, Sung et al. showed in a multivariate analysis that recipients who smoke prior to renal transplantation have a relative risk of 2.3 for graft loss [14]. In another study, recipients who had a history of cigarette smoking had decreased patient survival after transplantation with a magnitude of negative impact similar to that of diabetes [10], although smoking cessation by the recipient did appear to have a protective effect against graft loss [14].

While the consequences of smoking on the part of the graft recipient are well known, the effects of tobacco use by the donor are poorly understood, and there is limited data to guide the practitioner on the use of kidneys from smoking donors [10–15]. To our knowledge, only one other study has examined the effects of living donor smoking on donor and recipient renal function. Robert et al. showed using a univariate analysis that tobacco use was a significant risk factor for delayed graft function after renal transplantation but were unable to replicate this result using multivariate analysis [18]. In addition, the conclusions of the Robert study are limited by the use of cadaveric kidneys, leaving the question unanswered for living kidney donors.

Our study demonstrates that tobacco use by the donor is damaging to the kidney function of both donors and recipients. While the differences in absolute Cr levels were not significant, significant differences were observed with percent change in Cr, which is a more sensitive indicator of renal function than absolute Cr [19]. In addition, recipients of kidneys from donors who smoked had lower GFR at 1 year and most recent followup. The fact that the effect of donor smoking upon recipient function shows a dose response curve further strengthens the findings of this study (Figure 2). 

While our data suggest that the use of kidneys from SDs results in worse outcomes for both donors and recipients, it also shows that smoking cessation on the part of the donor can have a protective effect, as Cr levels fell much more sharply in SD-R's when the donor had stopped smoking than when the donor was actively smoking. In our study, 55% of the SD group had quit using tobacco, with an average period of cessation prior to donation of 10.7 years. Previous studies have also shown that smoking cessation by the graft recipient can be protective as well: Kasiske and Klinger evaluated recipients with a history of tobacco use and found that the decreased graft and patient survival associated with tobacco use returned to baseline if the recipients have a history of tobacco cessation >5 years prior to transplantation [12]. While smoking cessation by the donor, recipient, or both can be protective, transplantation outcomes remain best when neither donor nor recipient has any history of tobacco use.

The results of our study suggest that both current and past tobacco exposure may have a negative impact upon donor and recipient function. However, these findings must be viewed in light of the limitations of this study. Our data is retrospective in nature, with all the inherent biases associated with that study design. In addition, the sample sizes of our SD and SD-R groups are small (*n* = 29) and follow-up times limited, which does not allow us to determine the optimal period of tobacco cessation prior to kidney donation. Although a long period of time passed since our initial study period, we were unable to obtain additional followup for the donors, as many do not live locally and were only involved at our institution for a short time surrounding the operation. Furthermore, this study was not sufficiently powered to definitively determine whether donors with a tobacco history should be allowed to become living donors, as this would require additional data to establish whether receiving a graft from a smoking donor is better or worse than not receiving a graft at all. Finally, the short-term followup did not allow for the determination of long-term risk for renal insufficiency and renal failure in a donor who continues to smoke. Given these limitations, the results of our study will need to be validated by other centers before adopting any universal policy regarding the amount of tobacco use that would eliminate a potential donor from consideration. Until these studies are completed, donors with a significant history of smoking should be used cautiously and only after appropriate counseling with both the donor and recipient. 

## 5. Conclusions

Donors who actively smoke or have a past history of tobacco use have a larger percent increase in Cr at one year following donation compared to donors who have never smoked. Recipients of kidneys from SDs showed significantly less improvement in long-term postoperative Cr and lower GFR's when compared to recipients of ND kidneys. While smoking cessation by the donors can be protective, kidneys from donors with no history of smoking provide the best outcomes. Donors with prior tobacco exposure should be used with caution due to the negative impact upon both donor and recipient renal function.

## Figures and Tables

**Table 1:** Demographics and preoperative characteristics of 200 patients at a single institution, including 100 kidney donors undergoing laparoscopic donor nephrectomy as well as their 100 recipients. Patients were stratified by donor tobacco history into those with a history of tobacco use (SD) and a control group with no history of tobacco use (ND).

	SD	ND	*P*
*Donors*	*n* = 29	*n* = 71	
Mean age (years)	36.8	34.4	0.31
Mean BMI (kg/m^2^)	26.4	25.5	0.24
Sex	45% M	41% M	0.89
Hypertension	0	0	n/a
Diabetes	0	0	n/a

*Recipients*	*n* = 29	*n* = 71	
Mean age (years)	39.8	38.7	0.78
Mean BMI (kg/m^2^)	24.5	25.4	0.48
Sex	44.8% M	62.0% M	0.18
Hypertension	29 (100%)	66 (93%)	0.34
Diabetes	9 (31%)	15 (21%)	0.43

**Table 2:** Calculated glomerular filtration rates in mL/min per 1.73 m^2^ of 200 patients at a single institution, including 100 kidney donors undergoing laparoscopic donor nephrectomy as well as their 100 recipients. Patients were stratified by donor tobacco history into those with a history of tobacco use (SD) and a control group with no history of tobacco use (ND).

	SD	ND	*P*
*Recipients*	*n* = 29	*n* = 71	
Preoperative	7.1	6.8	0.67
1 week	66.5	59.5	0.14
1 month	62.8	60.4	0.60
6 months	53.7	54.9	0.77
1 year	44.1	54.7	**0.018**
Most recent	37.0	53.0	**<0.001**
Peak	77.1	79.4	0.70

*Donors*	*n* = 29	*n* = 71	
Preoperative	96.2	104.0	0.14
1 week	57.0	60.7	0.20
Most recent	57.7	62.8	0.11
Nadir	50.0	52.7	0.21
